# Poor Sleep Quality Decreases Concurrent Training Benefits in Markers of Metabolic Syndrome and Quality of Life of Morbidly Obese Patients

**DOI:** 10.3390/ijerph17186804

**Published:** 2020-09-18

**Authors:** Pedro Delgado-Floody, Pedro Ángel Latorre-Román, Daniel Jerez-Mayorga, Felipe Caamaño-Navarrete, Johnattan Cano-Montoya, José Alberto Laredo-Aguilera, Juan Manuel Carmona-Torres, Ana Isabel Cobo-Cuenca, Diana P. Pozuelo-Carrascosa, Cristian Álvarez

**Affiliations:** 1 Department of Physical Education, Sport and Recreation, Universidad de La Frontera, Temuco 4780000, Chile; pedro.delgado@ufrontera.cl; 2 Department of Didactics of Corporal Expression, University of Jaen, 23400 Jaen, Spain; platorre@ujaen.es; 3 Faculty of Rehabilitation Sciences, Universidad Andres Bello, Santiago 7591538, Chile; daniel.jerez@unab.cl; 4 Faculty of Education, Universidad Católica de Temuco, Temuco 4780000, Chile; marfel77@gmail.com; 5 School of Kinesiology, Faculty of Health Sciences, Universidad San Sebastian, Valdivia 5090000, Chile; jcanom@docente.uss.cl; 6 Multidisciplinary Research Group in Care (IMCU), Universidad de Castilla-La Mancha, 45004 Toledo, Spain; JoseAlberto.Laredo@uclm.es (J.A.L.-A.); JuanManuel.Carmona@uclm.es (J.M.C.-T.); AnaIsabel.Cobo@uclm.es (A.I.C.-C.); DianaP.Pozuelo@uclm.es (D.P.P.-C.); 7 Facultad de Fisioterapia y Enfermería de Toledo, Universidad de Castilla-La Mancha, 45004 Toledo, Spain; 8 Quality of Life and Wellness Research Group API4, Laboratory of Human Performance, Department of Physical Activity Sciences, Universidad de Los Lagos, Osorno 5290000, Chile

**Keywords:** morbid obesity, exercise, sleep quality, quality of life

## Abstract

**Background:** Sleep quality (SQ) plays a role in multiple activities of daily living, but little is known about its role in concurrent training [CT, high-intensity interval (HIIT) plus resistance training (RT)] adaptations for metabolic syndrome (MetS) and health-related quality of life (HRQoL) markers. The aim of the present study was to determine the effects of a 20-week CT programme on MetS and HRQoL markers according to the SQ of morbidly obese patients. **Methods:** Twenty-nine morbidly obese patients were allocated to one of two groups: good sleep quality (GSQ, n = 15, 38.07 ± 12.26 years) and poor sleep quality (PSQ, n = 14, 40.79 ± 11.62 years). HRQoL, body mass index, waist circumference (WC), systolic and diastolic blood pressure (SBP and DBP, respectively), and plasma outcomes were measured. **Results:** The GSQ group reported significant changes (pre- vs. post-intervention) in WC (114.0 ± 3.1 vs. 110.4 ± 3.4 cm, p = 0.012), SBP (137.0 ± 4.3 vs. 125.6 ± 1.8 mmHg, p = 0.006), and HRQoL general health (51.33 ± 21.08 vs. 64.33 ± 16.24, p = 0.020). By contrast, the PSQ group showed significant changes only in SQ (9.00 ± 2.42 vs. 5.36 ± 2.84, p = 0.004). **Conclusions:** Morbidly obese PSQ patients showed a lower response for improving MetS and HRQoL markers after a 20-week CT programme than GSQ peers. However, there was a greater effect size for decreasing WC and SBP in favour of the GSQ compared with the PSQ group, suggesting that there are limitations to CT benefits on these outcomes in the PSQ group. These results call for more complex future studies.

## 1. Introduction

Morbid obesity is a chronic disease with life-threatening potential consequences such as metabolic syndrome (MetS) [1]. This condition contributes to the impairment of all domains of health-related quality of life (HRQoL) [2]; it has been associated with an increase in sleep-disordered breathing conditions like obstructive sleep apnoea and obesity hypoventilation syndrome, both of which affect sleep quality [3]. In this sense, poor sleep quality (SQ) has been strongly associated with mood disturbance and poor quality of life among extremely obese patients [4].

Sleep is an important modulator of neuroendocrine function and glucose metabolism, and poor SQ is related to more metabolic and endocrine alterations, including decreased glucose tolerance, decreased insulin sensitivity, and increased hunger and appetite [5]. Other disruptions include that sleep is related to several other MetS risk factors [i.e., higher waist circumference (WC), hypertension, elevated serum triglycerides (TG), low serum high-density lipoprotein cholesterol (HDL-C), and hyperglycaemia)] [6]. Adopting a physically active lifestyle may be a key intervention to treat poor SQ in morbidly obese patients and may be a long-term solution for improving overall quality of life in this cohort [7].

Exercise training is recognised as a therapeutic tool for several chronic diseases [8]. Some of these benefits include prevention and treatment of MetS risk factors, improvement of strength and cardiorespiratory fitness [9], and also protection against premature cardiovascular death in several populations [10,11,12]. In this sense, resistance training (RT), defined as any exercise that causes voluntary skeletal muscle stimulation, generally using external weights such as dumbbells, bars, one’s own body weight, or any other exercise equipment [13], improves muscle strength and functional capacity. RT can improve daily living activities in obese patients before bariatric surgery [14]. High-intensity interval training (HIIT), defined as several brief bouts of high-intensity efforts (e.g., cycling or running) interspersed by recovery periods [15], has also been reported to improve risk factors for type 2 diabetes mellitus, hypertension, central arterial stiffness, vascular function, and cardiorespiratory fitness [16,17]. Thus, HIIT offers protective effects against the development of these cardiometabolic diseases [18,19]. Moreover, concurrent training (CT), which includes HIIT and RT, may help to improve cardiovascular outcomes such as reducing systolic blood pressure (SBP) and thus contribute to decreased mortality associated with cardiovascular disease [20].

Poor SQ alters the beneficial effects of exercise training adaptations [9]. Only part of the mechanism(s) underlying these poor-SQ-mediated effects on CT adaptations have been described; they may be related to the balance between hormones that increase and suppress hunger, where subjects who sleep less (i.e., have poor SQ) have more ghrelin, hunger, and appetite and less leptin compared with peers who sleep more [21]. At the cardiorespiratory level, poor SQ promotes several deficits in the nervous and endocrine systems: (a) decreased physical performance, (b) decreased concentration and memory, (c) more insulin resistance, and (d) decreased leptin (appetite suppressor). When there are several continuous sleepless nights, these systems are dysregulated [22]. Thus, the aim of this study was to determine the effects of a 20-week CT programme on MetS and HRQoL markers according to the SQ of morbidly obese patients.

## 2. Materials and Methods

### 2.1. Study Design

This study evaluated the effects of a 20-week CT programme in morbidly obese patients (2 times/week). The sample size was determined by the free availably online G*Power 3 software (Heinrich-Heine-Universität, Düsseldorf, Germany), using delta changes (∆) and standard deviations (SDs) from a similar exercise intervention extension and cohort (patients with obesity), where MetS outcomes (blood pressure) were included [23]. Based on one predictive outcome [SBP (SD = 6 mmHg)], a moderate effect size (0.60), and a critical *t* value of 1.73, 10 subjects per group would give 80% power with α < 0.05. The study was carried out in accordance with the Declaration of Helsinki (2013) and was approved by the Ethical Committee of the Universidad de La Frontera, Temuco, Chile (DI18-0043 Project).

### 2.2. Patients and Recruitment

The inclusion criteria were (i) being a candidate for bariatric surgery, (ii) age > 18 but < 60 years, (iii) medical authorisation, and (iv) body mass index (BMI) ≥ 35 kg/m^2^. The exclusion criteria were (i) physical limitations to performing the exercises (e.g., restrictive injuries of the musculoskeletal system), (ii) exercise-related dyspnoea or respiratory alterations, (iii) chronic heart disease with any worsening in the last month, and (iv) less than 80% adherence to the total intervention (excluded from the statistical analyses).

**Figure 1:**
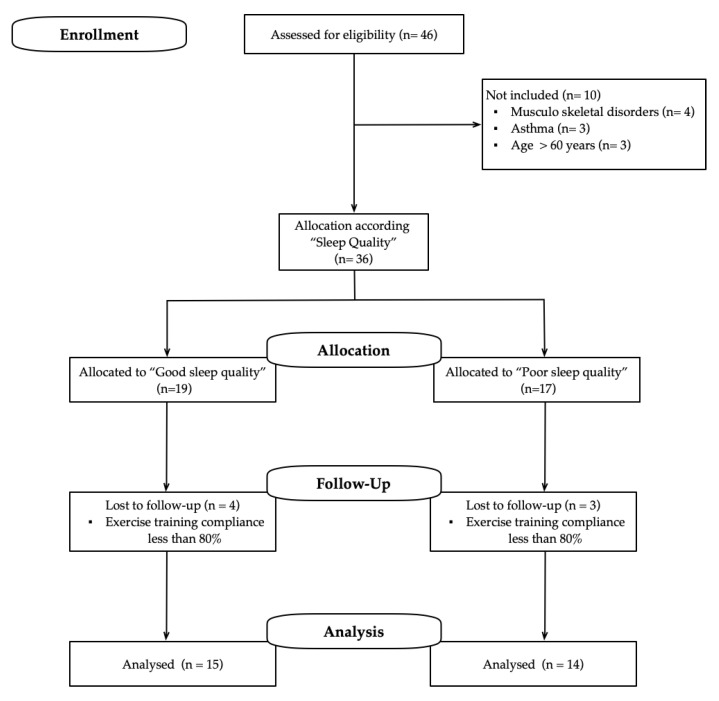
Study design.

After the enrolment stage, 46 participants were assessed for elig¡ibility; 10 did not meet the inclusion criteria. After follow-up loss (*n* = 7), 29 participants were allocated to one of the following groups according to their SQ: good-sleep-quality group (GSQ, *n* = 15, 38.0 ± 12.2 years, woman, *n* = 13) and poor-sleep-quality group (PSQ, *n* = 14, 40.7 ± 11.6 years, woman, *n* = 13). Figure 1 shows the study design.

### 2.3. Measurements

#### 2.3.1. SQ Measurements

SQ was assessed using the Pittsburgh Sleep Quality Index (PSQI) [24]. The PSQI is a self-report questionnaire that includes seven component scores (subjective SQ, sleep latency, sleep duration, habitual sleep efficiency, sleep disturbances, use of sleeping medication, and daytime dysfunction). In the PSQI, subjects rate perceived SQ as very good, fairly good, fairly bad, or very bad. These subjective scales are weighted to obtain a global PSQI score that differentiates between good and poor SQ. Their sum builds the global PSQI report, which provides an ‘inverse score’, where a score <5 denotes high SQ and a score >5 denotes low SQ. This scale has been used in previous studies [25] and publications that examined bariatric patients [26]. Conditions associated with PSQ included the use of sleep medications, difficulties in daily living and enthusiasm, and low sleep efficiency.

#### 2.3.2. MetS Markers

After overnight fasting (10–12 h), all patients underwent a baseline assessment (pre-test) between 08:00 h and 9:00 h in the morning. The following variables were assessed; fasting glucose, total cholesterol (TC), low-density lipoprotein cholesterol (LDL-C), HDL-C, and TG. SBP and diastolic blood pressure (DBP) were measured according to a previous publication [27]. WC was assessed with a tape measure graduated in centimetres (Adult SECA^TM^), following a standard protocol [28].

#### 2.3.3. HRQoL

HRQoL was measured with the Short Form-36 (SF-36) [29]. This questionnaire contains eight scales designed to evaluate physical health as well as mental functioning of the subject. The scales are (i) physical functioning, (ii) role physical, (iii) pain, (iv) general health, (v) vitality, (vi) social functioning, (vii) role emotional, and (viii) mental health. The subjects are asked to give answers on a numerical scale; those answers are then coded and assigned a score on a scale of 0–100; a higher score represents a better result in view of the subjective perception of physical and mental health.

#### 2.3.4. Body Composition and Anthropometrics Parameters

Body mass (kg) and body fat (% and kg) were measured using a digital bioimpedance scale (TANITA^TM^, model 331, Tokyo, Japan). Height (m) was measured with a SECA^TM^ stadiometer (model 214, Hamburg, Germany), with subjects in light clothing and without shoes. BMI was calculated as the body weight divided by the square of the height (kg/m^2^). BMI was determined to estimate the degree of obesity (kg/m^2^) using standard criteria for obesity and morbid obesity classification [30].

### 2.4. CT Intervention

The CT exercise programme had two components: RT and HIIT. First, the RT component included 3–4 (according to the planning week, Table 1) RT exercises that targeted the following muscle groups: forearm, knee flexors and extensors, trunk, chest, shoulder elevators, horizontal shoulder flexors and extensors, and plantar flexors. The exercises for each muscle group were performed in three 60 s sets (continuous concentric/eccentric voluntary contraction), followed by 60–120 s of passive recovery, as previously described [23]. To estimate the intensity of work in the different exercises of the programme, the maximum dynamic muscular strength (1RM) was estimated indirectly through the Brzycki formula [31], with fewer than 12 maximum repetitions. Second, the HIIT section was performed with 60 s of maximum-intensity exercise using a magnetic resistance static bicycle (Oxford^TM^ Fitness, model BE-2701, Santiago, Chile), followed by 60–120 s of passive recovery with the bicycle totally off; this cycle was repeated 4–7 times according to the weekly schedule [23]. The exercise intensity was based on the Borg scale of perceived exertion (from 1 to 10); the participants worked between 6 and 9 points. All sessions started with a 10 min warmup period with continuous walking and joint mobility and flexibility exercises, followed by 5 min of cooldown and stretching to prevent injuries. Table 1 shows the CT programme characteristics.

### 2.5. Data Analysis

Data are presented as the mean and SD in tables and as mean and standard error in figures. Normality and homoscedasticity assumptions for all data were analysed using the Shapiro–Wilk test and Levene’s test, respectively. Student’s *t*-test was used to identify differences at baseline. We also calculated the delta changes of each outcome and compared the adaptations between both groups. A two-way (group and time) repeated measures analysis of variance (ANOVA) was applied to assess the occurrence of an actual training effect (i.e., *p* < 0.05 for the group × time interaction by the different study outcomes). The Bonferroni post hoc test was applied for pairwise comparisons. In addition, Cohen’s *d* was used to detect effect size using the threshold values of ≤0.49, 0.50–0.79, and ≥0.80 for small, moderate, and large effects, respectively [32]. Analysis of covariance (ANCOVA) was applied to test those outcomes with different baseline values. All statistical analyses were performed with SPSS version 23.0 (SPSS^TM^ Inc., Chicago, IL, USA). For all analyses, *p ≤* 0.05 was considered statistically significant.

## 3. Results

### 3.1. Baseline Characteristics

At baseline (Table 2), there were significant differences between the GSQ and PSQ groups in BMI (GSQ, 40.1 ± 5.8 kg/m^2^; PSQ, 46.1 ± 7.0 kg/m^2^; *p* = 0.019) and body fat (GSQ, 46.2% ± 4.2%; PSQ, 50.2% ± 4.5%; *p* = 0.019).

### 3.2. Training-Induced Changes in MetS Markers

**Figure 2:**
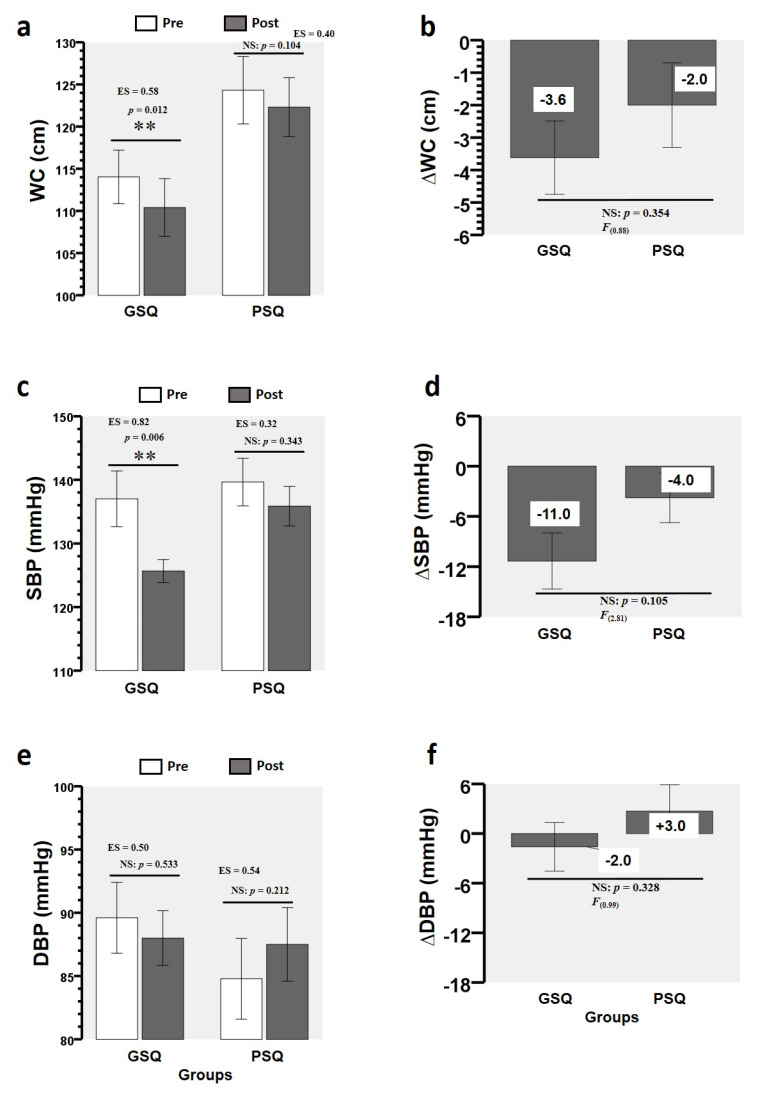
Changes in metabolic syndrome makers for each sleep-quality group. Abbreviations: DBP, diastolic blood pressure; GSQ, good sleep quality; PSQ, poor sleep quality; WC, waist circumference. (∆) denotes delta changes from pre to post intervention. (**) denotes significant differences between groups (*p* < 0.01). (NS) denotes no significant differences. (ES) denotes effect size. (F) denotes the Levene test. After the CT programme, there was a change in the GSQ group between pre- and post-intervention WC (114.0 ± 3.1 vs. 110.4 ± 3.4 cm, ES = 0.58, *p* = 0.012), but no change in the PSQ group (*p* = 0.104) (**a**). There was a difference in the pre- and post-intervention SBP for the GSQ group (137.0 ± 4.3 vs. 125.6 ± 1.8 mmHg, ES = 0.82, *p* = 0.006) but not for the PSQ group (*p* = 0.343) (**c**). There were no differences in pre- and post-intervention DBP for the GSQ (*p* = 0.533) and PSQ (*p* = 0.212) groups (**e**). There were no time × group interactions for the ∆WC (**b**), ∆SBP (**d**), and ∆DBP (**f**) comparisons between groups.

After the CT programme, there was a change in the GSQ group between pre- and post-intervention WC (114.0 ± 3.1 vs. 110.4 ± 3.4 cm, ES = 0.58, *p* = 0.012), but no change in the PSQ group (*p* = 0.104) (Figure 2a). There was a difference in the pre- and post-intervention SBP for the GSQ group (137.0 ± 4.3 vs. 125.6 ± 1.8 mmHg, ES = 0.82, *p* = 0.006) but not for the PSQ group (*p* = 0.343) (Figure 2c). There were no differences in pre- and post-intervention DBP for the GSQ (*p* = 0.533) and PSQ (*p* = 0.212) groups (Figure 2e). There were no time × group interactions for the ∆WC (Figure 2b), ∆SBP (Figure 2d), and ∆DBP (Figure 2f) comparisons between groups.

**Figure 3:**
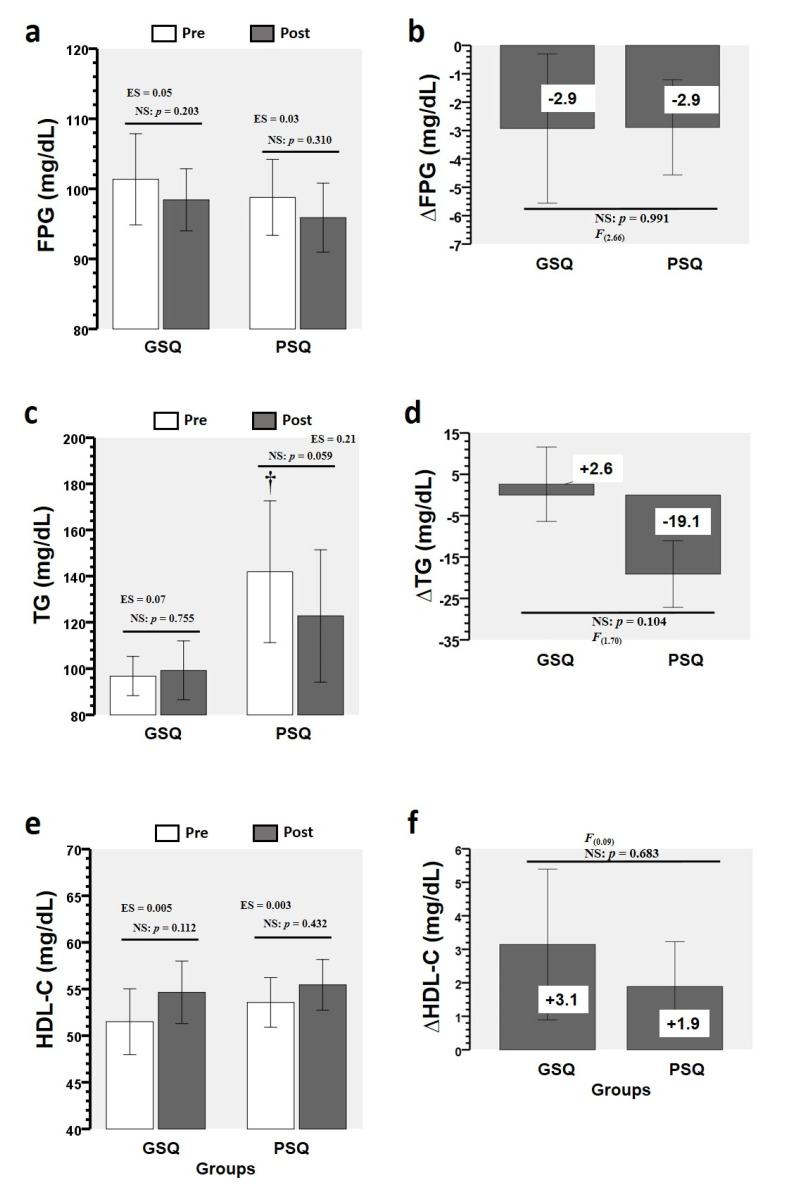
Changes in metabolic syndrome makers for each sleep-quality group. (∆) denotes delta changes from pre to post intervention. Abbreviations: FPG, fasting plasma glucose; GSQ, good sleep quality; HDL-C, high-density lipoprotein cholesterol; LDL-C, low-density lipoprotein cholesterol; PSQ, poor sleep quality; TC, total cholesterol; TG, triglycerides. (†) denotes significant baseline differences (*p* < 0.05). (NS) denotes no significant differences. (ES) denotes effect size. (F) denotes the Levene test. Comparing pre- and post-intervention values in GSQ and PSQ groups, there were no changes in FPG, TG, and HDL-C measures (**a**,**c**,**e**), and there were no time × group interactions for the ∆FPG (**b**), ∆TG (**d**), and ∆HDL-C (**f**) comparisons.

Comparing pre- and post-intervention values in GSQ and PSQ groups, there were no changes in FPG, TG, and HDL-C measures (Figure 3a,c,e), and there were no time × group interactions for the ∆FPG (Figure 3b), ∆TG (Figure 3d), and ∆HDL-C (Figure 3f) comparisons.

### 3.3. Training-Induced Changes in SQ and HRQoL

**Figure 4:**
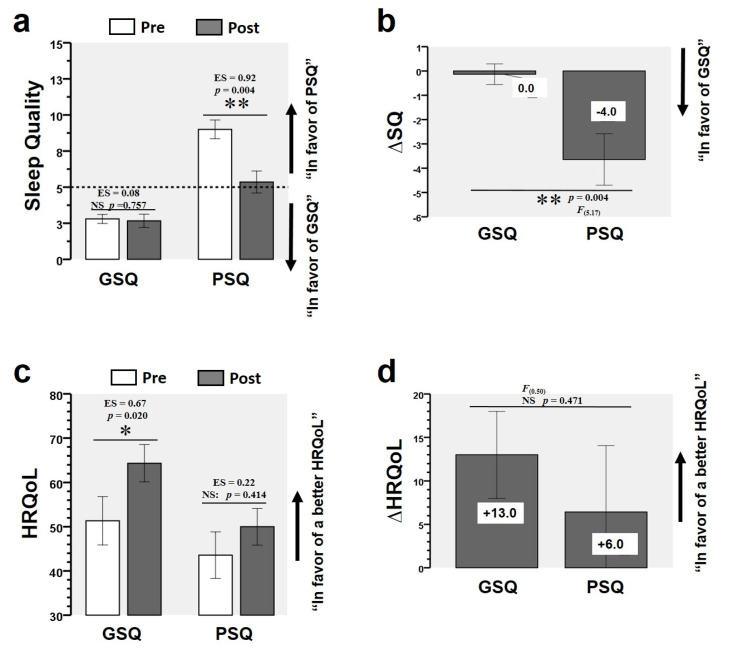
Changes in sleep quality (SQ) and in health-related quality of life (HRQoL) for the good-sleep-quality group (GSQ) and poor-sleep-quality (PSQ) group. (∆) denotes delta changes from pre to post intervention. (ES) denotes effect size. (F) denotes the Levene test. (*) denotes significant differences between groups (*p* < 0.05). (**) denotes significant differences between groups (*p* < 0.01). (NS) denotes no significant differences. In the GSQ group, there were no changes in the pre- and post-intervention SQ scores (*p* = 0.757) (**a**). By contrast, the PSQ group showed significant changes between pre- and post-intervention values (9.00 ± 2.42 vs. 5.36 ± 2.84, ES = 0.92, *p* = 0.004) (**a**). Comparing pre- and post-intervention in the GSQ group, there were changes in the HRQoL general health score (51.33 ± 21.08 vs. 64.33 ± 16.24, ES = 0.67, *p* = 0.020) (**c**). By contrast, the PSQ group showed no significant changes between pre- and post-intervention (*p* = 0.414) (**c**). There was a time × group interaction in ΔSQ between the GSQ and PSQ groups (Δ 0.0 vs. Δ −4.0, *F* = 0.50 *p* = 0.004) (**b**). There was no time × group interaction in the HRQoL between the GSQ and PSQ groups (**d**).

In the GSQ group, there were no changes in the pre- and post-intervention SQ scores (*p* = 0.757) (Figure 4a). By contrast, the PSQ group showed significant changes between pre- and post-intervention values (9.00 ± 2.42 vs. 5.36 ± 2.84, ES = 0.92, *p* = 0.004) (Figure 4a). Comparing pre- and post-intervention in the GSQ group, there were changes in the HRQoL general health score (51.33 ± 21.08 vs. 64.33 ± 16.24, ES = 0.67, *p* = 0.020) (Figure 4c). By contrast, the PSQ group showed no significant changes between pre- and post-intervention (*p* = 0.414) (Figure 4c). There was a time × group interaction in ΔSQ between the GSQ and PSQ groups (Δ 0.0 vs. Δ −4.0, *F* = 0.50 *p* = 0.004) (Figure 4b). There was no time × group interaction in the HRQoL between the GSQ and PSQ groups (Figure 4d).

## 4. Discussion

The present study aimed to determine the effects of a 20-week CT programme on MetS and HRQoL markers according to the SQ of morbidly obese patients. The main finding of this study is that PSQ morbidly obese patients showed no significant changes in the MetS markers WC and SBP, while GSQ peers showed significant improvements in these measures after CT intervention. This result is consistent with previously described findings from the literature: (a) the GSQ group had significant changes in WC and SBP and HRQoL after the CT intervention, (b) the PSQ group improved their SQ, and (c) although there were no differences between groups (GSQ vs. PSQ), the GSQ group presented a higher effect size than the PSQ group in WC and in SBP (medium and large effect size, respectively).

In the present study, the GSQ but not the PSQ group improved in MetS markers (specifically WC and SBP). Poor SQ is related to more metabolic alterations, including decreased glucose tolerance, decreased insulin sensitivity, and increased hunger and appetite [5]. These disturbances may affect the capacity of CT to promote beneficial adaptations in the PSQ group. In addition, poor SQ has been associated with hypertension in middle-aged adults, in whom it is well established that SQ alters autonomic nervous system function and other physiologic events that influence SBP [33].

The CT benefits in patients with MetS have been previously shown [34]. Barnes et al. [35] reported a WC reduction (19.6 cm) in patients with obstructive sleep apnoea (i.e., PSQ) after a 16-week CT programme (endurance and RT), but this intervention included a higher volume of exercise training per week compared with the present study. Moreover, in an RT intervention in morbidly obese patients without information about SQ, Delgado-Floody et al. [36] reported a ~5 mmHg reduction in ∆SBP; these values, although positive, are far from the reduction achieved in the present study in the GSQ group (∆SBP −11.3 mmHg). This difference could be explained by the combined nature of the training protocol carried out and the duration of the session (HIIT + RT vs. RT alone) [37]. By contrast, another investigation that evaluated the response to the exercise in MetS risk factors in morbidly obese adolescents with PSQ did not report significant changes after a 12-week CT programme (endurance + RT) [38], findings that are consistent with the present study.

With regard to the metabolic response to the CT intervention, there was a positive tendency—albeit not statistically significant—for a decrease in fasting plasma glucose and TG and an increase in HDL-C in the post-intervention measurements. In contrast with our results, Goodpaster et al. [39] reported favourable changes in MetS markers in patients with severe obesity after a 1-year intensive lifestyle intervention (i.e., diet and physical activity). In addition, Barnes et al. [35] reported a reduction in total cholesterol, LDL-C, TG, fasting plasma glucose, SBP/DBP, and BMI after a 16-week exercise and diet programme in similarly obese patients with sleep disorders. The reduced sleep duration has been paralleled by a trend of an increase in obesity [40]. Poor SQ has been associated with an increased risk of heart disease and mortality [41]. In this sense, Mendelson et al. [38] demonstrated that obese adolescents had poorer SQ than healthy-weight subjects; moreover, the authors reported that after exercise training, the obese group increased their sleep duration and sleep efficiency. Another study reported similar benefits: participation in a 6-month endurance exercise training programme improved SQ in obese patients with sleep disorders [42]. A recent study reported that variation in sleep duration and less physical activity were associated with a less favourable metabolic profile [43]. By contrast, another study conducted in Korean adolescents showed that short sleep duration was associated with overweight; however, the authors reported that there was no association between sleep duration and MetS [40].

In the present study, the PSQ group presented improved SQ after 20 weeks of CT. It has been observed that weight-loss interventions improve SQ [44]. An investigation reported improved SQ in morbidly obese patients with sleep disturbances after 16 weeks of CT [35]. Mendelson et al. [38] also reported improvements in sleep quality and quantity of sleep, but not in MetS markers, in morbidly obese adolescents with sleep disturbances after 12 weeks of CT. Jurado et al. [45] reported that 12-weeks of exercise intervention improved subjective SQ in adults. Moreover, the authors indicated that exercise holds promise as a non-pharmacological intervention for SQ improvement.

After the CT intervention, the GSQ but not the PSQ group showed improved HRQoL (general health dimension). Another study reported that a multidisciplinary treatment programme (i.e., caloric restriction and physical activity) improved HRQoL in obese youth [46]. Hageman et al. [47] demonstrated that weight loss is associated with improved HRQoL after a lifestyle intervention. Likewise, a recent study showed that poor SQ was associated with reduced HRQoL [48]. In this context, a study conducted with obese subjects reported that poor SQ negatively influences body composition, macronutrient intake, and HRQoL [49]. Therefore, early detection of sleep problems in obese subjects could prevent the potential development of psychological disorders [4].

### Strengths and Limitations

The main limitation of this study was that the participants’ food habits were not measured during the intervention; however, each week, the participants were reminded not to change their baseline eating patterns. By contrast, a strength of this study was that we included MetS as well as HRQoL and SQ markers, which are relevant as indicators of potential risk due to technological advancements, considering that spending a lot of time on electronic devices is a major reason why people may stay up later at night and get less sleep.

## 5. Conclusions

In the present study, morbidly obese patients with PSQ showed less improvements in the evaluated MetS and HRQoL markers after a 20 week CT programme compared with GSQ peers. These findings suggest that there are limitations that prevent CT from fully providing beneficial adaptations to the evaluated measures in the PSQ group. These results warrant more complex future studies to corroborate and expand this study.

## Figures and Tables

**Table 1:** Characteristics of the concurrent training programme (2 times/week).

	RT						HIIT				
Weeks	Intensity (%1RM)	Reps	Time (min)	Sets	Rest (min)	E N°	Intensity perception	Time (min)	Sets	Rest (min)	Duration (min)
1–2	40	20–25	1	3	2.0	3	6–7	1	4	2.0	45
3–4	40–50	25–30	1	3	2.0	3	6–7	1	4	2.0	45
5–6	45–50	30	1	3	1.5	4	6–7	1	5	1.5	45
7–8	45–50	30–35	1	3	1.5	4	7–8	1	5	1.5	45
9–10	50–55	25–30	1	3	1.5	4	7–8	1	5	1.5	45
11–12	50–55	25–30	1	3	1.5	4	7–8	1	6	1.5	45
13–14	50–55	30	1	3	1.0	4	7–8	1	6	1.0	45
15–16	50–55	30–35	1	3	1.0	4	8	1	6	1.0	45
17–18	55	30–35	1	3	1.0	4	8	1	7	1.0	45
19–20	55–60	30–35	1	3	1.0	4	8–9	1	7	1.0	45

1RM, maximum dynamic muscular strength; E N°, exercise number; RT, resistance training; HIIT, high-intensity interval training.

**Table 2:** Characteristics of the sample according to sleep quality.

Outcomes		Good Sleep Quality	Poor Sleep Quality	Good vs. Poor Sleep Quality (Baseline)	Good vs. Poor Sleep Quality (∆Pre–Post)
*n*		15	14		
Age (years)		38.0 ± 12.2	40.7 ± 11.6		
Height (m)		1.58 ± 0.07	1.59 ± 0.09		
Bedtime		22:32	22:45		
Time to fall asleep (min)		69.64	45.65		
Wake time		07:50	07:28		
Body mass (kg)	Pre	101.2 ± 19.5	116.8 ± 22.0	*p* = 0.190	*p* = 0.667
	Post	99.4 ± 18.9 *	114.6 ± 21.6 *		
	*p*	0.045	0.018		
	Δ	−1.7	−2.21		
	ES	0.09	0.10		
BMI (kg/m^2^)	Pre	40.1 ± 5.8	46.1 ± 7.0	*p* = 0.019 ^#^	*p* = 0.667
	Post	39.4 ± 5.6 *	45.2 ± 6.6 **		
	*p*	0.049	0.015		
	Δ	−0.71	−0.92		
	ES	0.12	0.13		
Body fat (%)	Pre	46.2 ± 4.2	50.2 ± 4.5	*p* = 0.019 ^#^	*p =*0.332
	Post	46.4 ± 4.0	49.9 ± 4.8		
	*p*	0.550	0.437		
	Δ	0.23	−0.31		
	ES	0.06	0.07		
Body fat (kg)	Pre	47.1 ± 11.5	59.4 ± 15.1	*p* = 0.020 ^#^	*p* = 0.306
	Post	46.7 ± 11.4	58.0 ± 15.1		
	*p*	0.468	0.040 *		
	Δ	−0.45	−1.38		
	ES	0.04	0.09		

Data represent the mean ± standard deviation. (∆) denotes delta changes from pre to post intervention. (*) denotes significant pre–post differences (*p* ≤ 0.05). (**) denotes significant pre–post differences (*p* ≤ 0.001). (#) denotes significant differences at baseline (*p* < 0.05). (y) Delta changes were compared by Student’s *t*-test. (ES) Cohen’s *d*, which denotes effect size; ≤0.49 denotes a small effect size; 0.50–0.79 denotes a medium effect size; and ≥0.80 denotes large effect size.
